# Supplementary material for: Characterization of an early-diverging KCNE potassium-channel auxiliary subunit in the jawless vertebrate lamprey
Source: eLife. 2026 Aug 26;15:RP111781. doi: 10.7554/eLife.111781 (PMC13516689; doi:10.7554/eLife.111781)
Supplement: Supplementary file 2 [file elife-111781-fig1-data1.zip › eLife-RP-RA-2026-111781R1_Figure1-source data1.pdf]

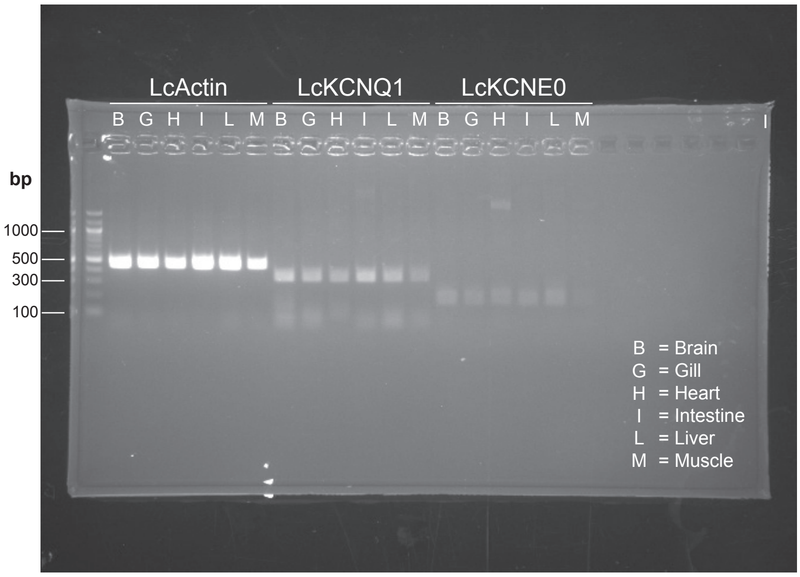

bp
1000
500
300
100
LcActin
LcKCNQ1
LcKCNE0
B G H I L M B G H I L M B G H I L M
B = Brain
G = Gill
H = Heart
I = Intestine
L = Liver
M = Muscle
