# Supplementary material for: Characterization of an early-diverging KCNE potassium-channel auxiliary subunit in the jawless vertebrate lamprey
Source: eLife. 2026 Aug 26;15:RP111781. doi: 10.7554/eLife.111781 (PMC13516689; doi:10.7554/eLife.111781)
Supplement: Peer review history

# Editor report: eLife Assessment

**DOI:** 10.7554/eLife.111781.3.sa0

**Authors:** Leon D Islas

**Affiliations:** Universidad Nacional Autónoma de México Mexico

**Keywords:** Convincing, Important

The authors describe a new member of the KCNE auxiliary subunits of potassium channels from a lamprey. This new subunit represents an early evolutionary member which confers new properties when expressed along with KCNQ channels. In the revised version of the manuscript, the authors present **convincing** evidence from several experimental approaches. The contents of this manuscript are **important** and should be relevant to understanding both the mechanism of modulation of KCNQ channels by KCNE subunits and the evolutionary history of these subunits, which this manuscript now extends to the divergence of early vertebrates.

# Referee report: Reviewer #1 (Public review)

**DOI:** 10.7554/eLife.111781.3.sa1

[Editors' note: this version has been assessed by the Reviewing Editor without further input from the original reviewers. The authors have addressed the comments raised in the previous round of review.]

Summary:

In this study, the authors describe an early diverging vertebrate KCNE gene present in jawless lampreys that they denote KCNE0.

Three forms of the protein are isolated from different lampreys, which have 95% homology to each other, but only moderate homology to KCNE1-6.

Co-expression with lamprey KCNQ1 produced a non-inactivating current, whereas co-expression with mammalian KCNQ1 resulted in less modulation. Introduction of a tetra-leucine motif from KCNE4 into KCNE0 reduced current on co-expression with KCNQ1, conferring an inhibitory effect.

Strengths:

This is an interesting and uncontroversial report of a new KCNE isoform from lower vertebrates that gives insight into the evolutionary progression of the sequence and functional properties of the accessory protein.

# Referee report: Reviewer #2 (Public review)

**DOI:** 10.7554/eLife.111781.3.sa2

Summary:

This study functionally characterizes a single KCNE-like gene, kcne0, from a jawless vertebrate. The authors conducted multiple experiments, including TEVC, VCF, RT-PCR, and RNA-seq to show that KCNQ1 and kcne0 exhibited a broadly overlapping organ distribution in lamprey species, and KCNE0 produced a constitutively active current when co-expressed with lamprey KCNQ1, similar to the effects of human KCNE3 on KCNQ1. This modulation was species-specific, as co-expression of KCNE0 with other species' KCNQ1 was less effective. Moreover, the authors found that truncating the N-terminal had a more significant reduction of the modulatory effects than truncating the C-terminal of KCNE0. Interestingly, the introduction of the tetra-leucine motif from human KCNE4 into KCNE0 conferred KCNE0 with comparable effects of human KCNE4 on KCNQ1.

Strengths:

The authors clearly introduced an early-diverging member of the KCNE family, and convincingly demonstrated the function of this gene, KCNE0. The results are supported by experiments of multiple approaches and are clearly written. The work is significant and will interest readers from the extended research area.

Weaknesses:

No major concerns were identified with the manuscript in general.

# Author comment: Author response

**DOI:** 10.7554/eLife.111781.3.sa3

**Authors:** Go Kasuya, Kaei Ryu, Buntaro Zempo, Emi Kawano-Yamashita, Koichi Nakajo

**Affiliations:** Jichi Medical Univesity Shimotsuke Japan; Jichi Medical University Shimotsuke Japan; Jichi Medical University Shimotsuke-shi, Tochigi Japan; Nara Women’s University Nara Japan; Jichi Medical University Shimotsuke Japan

The following is the authors’ response to the original reviews.

**Public Reviews:**

**Reviewer #1 (Public review):**

Summary:

In this study, the authors describe an early diverging vertebrate KCNE gene present in jawless lampreys that they denote KCNE0.

Three forms of the protein are isolated from different lampreys, which have 95% homology to each other, but only moderate homology to KCNE1-6.

Co-expression with lamprey KCNQ1 produced a non-inactivating current, whereas co-expression with mammalian KCNQ1 resulted in less modulation. Introduction of a tetra-leucine motif from KCNE4 into KCNE0 reduced current on co-expression with KCNQ1, conferring an inhibitory effect.

Strengths:

This is an interesting and uncontroversial report of a new KCNE isoform from lower vertebrates that gives insight into the evolutionary progression of the sequence and functional properties of the accessory protein.

Thank you for reviewing our manuscript and for your constructive comments. Our point-to-point responses are shown below.

Weaknesses:

(1) No error bars visible for lamprey Q1 isoforms (open symbols) in Figure 2G. No statistical comparison was provided to indicate whether lamprey Q1 isoform V1/2s are significantly different (nor in Supplementary Table 1).

(2) There is the same issue in Figures 3 and 4. No appropriate statistical comparison is made between V1/2s for different truncations of PmKCNE0 (Figure 3), or between KCNQ1 species isoforms with and without PmE0.

We thank you for these helpful comments. Based on your suggestions, we revised the presentation of error bars in Fig. 2G and in other panels showing G–V or F–V relationships (Figs. 2J, 3F, 3N, 4C, 4F, 4I, 4L, and 5E; Supplementary Fig. 5D) to make the SEM bars clearer. We also added statistical comparisons of *V_1/2_* values among the three lamprey KCNQ1 orthologs in Fig. 2G and among truncation-series constructs in Figs. 3F and 3N using one-way ANOVA followed by Tukey–Kramer multiple-comparison tests. For Fig. 4, we added statistical comparisons between KCNQ1 species isoforms expressed with or without PmKCNE0 (Figs. 4C, 4F, and 4I), and between PmKCNQ1 expressed alone or with human KCNE1 or KCNE3 (Fig. 4L), using unpaired two-tailed Welch’s t-tests. These statistical comparisons are included in Supplementary Table 1.

**Reviewer #2 (Public review):**

Summary:

This study functionally characterizes a single KCNE-like gene, kcne0, from a jawless vertebrate. The authors conducted multiple experiments, including TEVC, VCF, RT-PCR, and RNA-seq to show that KCNQ1 and kcne0 exhibited a broadly overlapping organ distribution in lamprey species, and KCNE0 produced a constitutively active current when co-expressed with lamprey KCNQ1, similar to the effects of human KCNE3 on KCNQ1. This modulation was species-specific, as co-expression of KCNE0 with other species' KCNQ1 was less effective. Moreover, the authors found that truncating the N-terminal had a more significant reduction of the modulatory effects than truncating the C-terminal of KCNE0. Interestingly, the introduction of the tetra-leucine motif from human KCNE4 into KCNE0 conferred KCNE0 with comparable effects of human KCNE4 on KCNQ1.

Strengths:

The authors clearly introduced an early-diverging member of the KCNE family, and convincingly demonstrated the function of this gene, KCNE0. The results are supported by experiments of multiple approaches and are clearly written. The work is significant and will interest readers from the extended research area.

Weaknesses:

No major concerns were identified with the manuscript in general.

We thank you for the positive assessment of our work and for the constructive suggestions. Our point-by-point responses are provided below.

**Recommendations for the authors:**

**Reviewer #2 (Recommendations for the authors):**

(1) What is the physiological role of this KCNE0 and lamprey KCNQ1 in the lamprey species? While the authors mention that the physiological roles of KCNE0 are the next focus, it is preferable to discuss some of the potential functional significance of this newly characterised KCNE.

We thank you for this helpful suggestion. We agree that discussing the potential physiological roles of KCNQ1–KCNE0 complexes in lamprey strengthens the manuscript. We have therefore expanded the Discussion to raise the possibility that, given the broad tissue distribution of *kcne0* transcripts and the ability of KCNE0 to render lamprey KCNQ1 constitutively active, KCNQ1–KCNE0 complexes may contribute to general ion homeostasis, potentially analogous to the epithelial K^+^ recycling function of mammalian KCNQ1–KCNE3, rather than to the highly specialized KCNQ1–KCNE1 function in the mammalian heart and inner ear (page 14, lines 263–267).

(2) Human KCNQ1 has 676 amino acids, but LcKCNQ1 contains just 507 amino acids. The species-specific regulatory effects of KCNE0 may not only be attributed to KCNE0 itself but might also be influenced by the species of KCNQ1. Some discussion on this possibility will be helpful.

We thank you for raising this important point. We agree that the species-specific regulatory effects observed in our cross-species pairing experiments are unlikely to be determined by KCNE0 alone and may also be influenced by species-specific features of the KCNQ1 α-subunit. To address this point, we expanded the Discussion to note that the KCNQ1 proteins used in this study vary in amino-acid length, largely reflecting differences in the cytoplasmic C-terminal region, which may affect KCNQ1–KCNE compatibility and thereby influence channel gating and coupling to KCNE subunits (pages 12–13, lines 227–238). We also updated Supplementary Fig. 4 to include LrKCNQ1 and LcKCNQ1, and clarified that the LcKCNQ1 construct used in this study encodes 644 amino acids.

(3) In Supplementary Figure 3, bands corresponding to LcKCNQ1 (507 amino acids, Supplementary Figure 1) were not seen.

We thank you for pointing out this potentially confusing point. Supplementary Fig. 3 shows RT-PCR products amplified from tissue cDNA, not full-length amplification of the LcKCNQ1 ORF. As stated in the Methods section (pages 19–20, lines 374–399), the primers used for RT-PCR in Fig. 1F and Supplementary Fig. 3 were different from those used for cloning the full-length LcKCNQ1 cDNA shown in Supplementary Fig. 1. Therefore, a band corresponding to the full-length LcKCNQ1 coding sequence was not expected in Supplementary Fig. 3.

To clarify this point, we revised the figure legends and indicated the RT-PCR primer-binding sites with orange arrows in Supplementary Figs. 1 and 2.
